# Barriers and enabling factors in weight management of patients with nonalcoholic fatty liver disease: A qualitative study using the COM‐B model of behaviour

**DOI:** 10.1111/hex.13665

**Published:** 2022-11-16

**Authors:** Yunpeng Gu, Run Zhou, Tingting Kong, Wei Zhang, Yutong Chen, Chunmei Wang, Junping Shi, Yanli Hu

**Affiliations:** ^1^ School of Public Health, Division of Health Sciences Hangzhou Normal University Zhejiang Hangzhou China; ^2^ School of Nursing, Division of Health Sciences Hangzhou Normal University Zhejiang Hangzhou China; ^3^ The Department of Metabolic Diseases Center the Affiliated Hospital of Hangzhou Normal University Zhejiang Hangzhou China; ^4^ The Department of Hepatology The Affiliated Hospital of Hangzhou Normal University Hangzhou Zhejiang China; ^5^ School of Nursing, Division of Medicine Jinan University Guangdong Guangzhou China

**Keywords:** COM‐B model, health care, nonalcoholic fatty liver disease, qualitative study, weight management

## Abstract

**Background:**

Nonalcoholic fatty liver disease (NAFLD) is a global public health problem. Lifestyle modifications aimed at promoting weight loss and weight maintenance remain the current first‐line treatments for NAFLD.

**Objective:**

We aim to identify barriers and enabling factors in weight management among patients with NAFLD using the capability, opportunity, motivation, behaviour (COM‐B) model of behaviour.

**Design:**

This study adopted a qualitative design using semistructured interviews analysed with content analysis and the COM‐B framework.

**Setting and Participants:**

Interviews were conducted with 27 patients with NAFLD who experienced successful or unsuccessful weight reduction.

**Results:**

Our study included 27 participants: 15 participants with successful weight loss (successful weight loss refers to a decrease in body weight ≥7% of the initial body weight for patients with NAFLD) and 12 participants with unsuccessful weight loss. Thirty‐five themes (19 barriers and 16 facilitators) were mapped onto the COM‐B model as barriers and facilitators to weight management among patients with NAFLD. The key barriers were lack of time and energy, lack of awareness of weight, lack of attention to NAFLD, treating food as a reward or compensation and social entertainment. The key facilitators were having basic weight loss knowledge and skills, strong motivation, attention to NAFLD, unsuccessful weight loss experiences and positive feedback from phased success.

**Conclusion:**

In addition to identifying factors consistent with existing studies, this study identified factors that influence weight management in NAFLD patients, such as basic weight loss skills and rational thinking before weight loss, which were not previously reported. This has clinical implications for clinical healthcare providers and health management services for the improvement of education and support regarding lifestyle improvement and weight management in patients with NAFLD.

**Patient or Public Contribution:**

We recruited potential participants from the Bariatric Clinic, Hepatology Clinic and Physical Examination Center of hospitals between March 2021 and October 2021. Twenty‐seven patients with NAFLD who had successful or unsuccessful weight loss experiences participated in the study and responded to questions on weight management.

## 1 INTRODUCTION

Nonalcoholic fatty liver disease (NAFLD) is a progressive disease that includes a spectrum of histopathology ranging from steatosis to nonalcoholic steatohepatitis (NASH), with a risk of progressive fibrosis that may lead to cirrhosis and hepatocellular carcinoma.^1^ As the incidence of obesity increases, NAFLD has become the primary cause of chronic liver disease, with a high incidence rate of up to 25% worldwide.^2^ Strikingly, the prevalence of NAFLD in China has increased substantially from 18% to 29.2% within a decade.^3^, ^4^ China is predicted to have the fastest‐growing NAFLD epidemic in the world and will reach 314.58 million patients by 2030, accompanied by a substantial economic burden and represents a growing public health problem.^5^

The increased global prevalence of NAFLD is particularly worrisome. Although no single recognized treatment is available, progress has been achieved in this field, and weight loss and weight maintenance remain the mainstay of treatment.^6^, ^7^ Paired liver biopsy studies have revealed that body weight loss ≥5% in patients with NAFLD is associated with a significant reduction in the incidence of hepatic steatosis (HS), weight loss ≥7% decreases the risk of hepatic inflammation and weight loss ≥10% decreases the risk of liver fibrosis.^8^ Unfortunately, although the significance of weight management for patients with NAFLD has been recognized by experts and doctors, the management of patients' weights is still not adequately implemented.^9^, ^10^, ^11^, ^12^ Possible explanations include the lack of implementation of behavioural programmes, lack of funds, clinicians' hesitancy to offer support and other factors. Guidance from theory might contribute to addressing these issues.

The capability, opportunity, motivation, behaviour (COM‐B) model is one theory of behaviour change proposed in 2011 by Michie et al.^13^ Capability is defined as an individual's psychological and physical capacity to engage in the targeted activity. Motivation is defined as the brain processes that energize and direct behaviour. Opportunity is defined as the factors external to the individual that enable or prompt the behaviour.^13^ These components interact to generate behaviour that in turn influences these components. The advantage of the COM‐B model is that it provides a useful framework to explain the barriers and enabling factors of behaviour change and provide a basis for the design of behavioural interventions. Therefore, this model has been applied to a number of clinical problems,^14^, ^15^, ^16^, ^17^ but it has not yet been applied to weight management in NAFLD patients. The aim of this study was to use the COM‐B model to describe barriers and enabling factors to weight loss from the perspective of patients with NAFLD who have experienced weight loss.

## 2 METHODS

We report this study following the Consolidated Criteria for Reporting Qualitative Research guidelines.^18^

### 2.1 Study design

We used a purposeful sampling approach to include participants of different genders, ages, cultures and economic conditions. We performed a qualitative study that included semistructured interviews with patients with NAFLD who experienced successful or unsuccessful weight reduction. The study was approved by the Ethics Committee of the Affiliated Hospital of Hangzhou Normal University (2022E2‐HS‐033).

### 2.2 Participants

**Figure 1:**
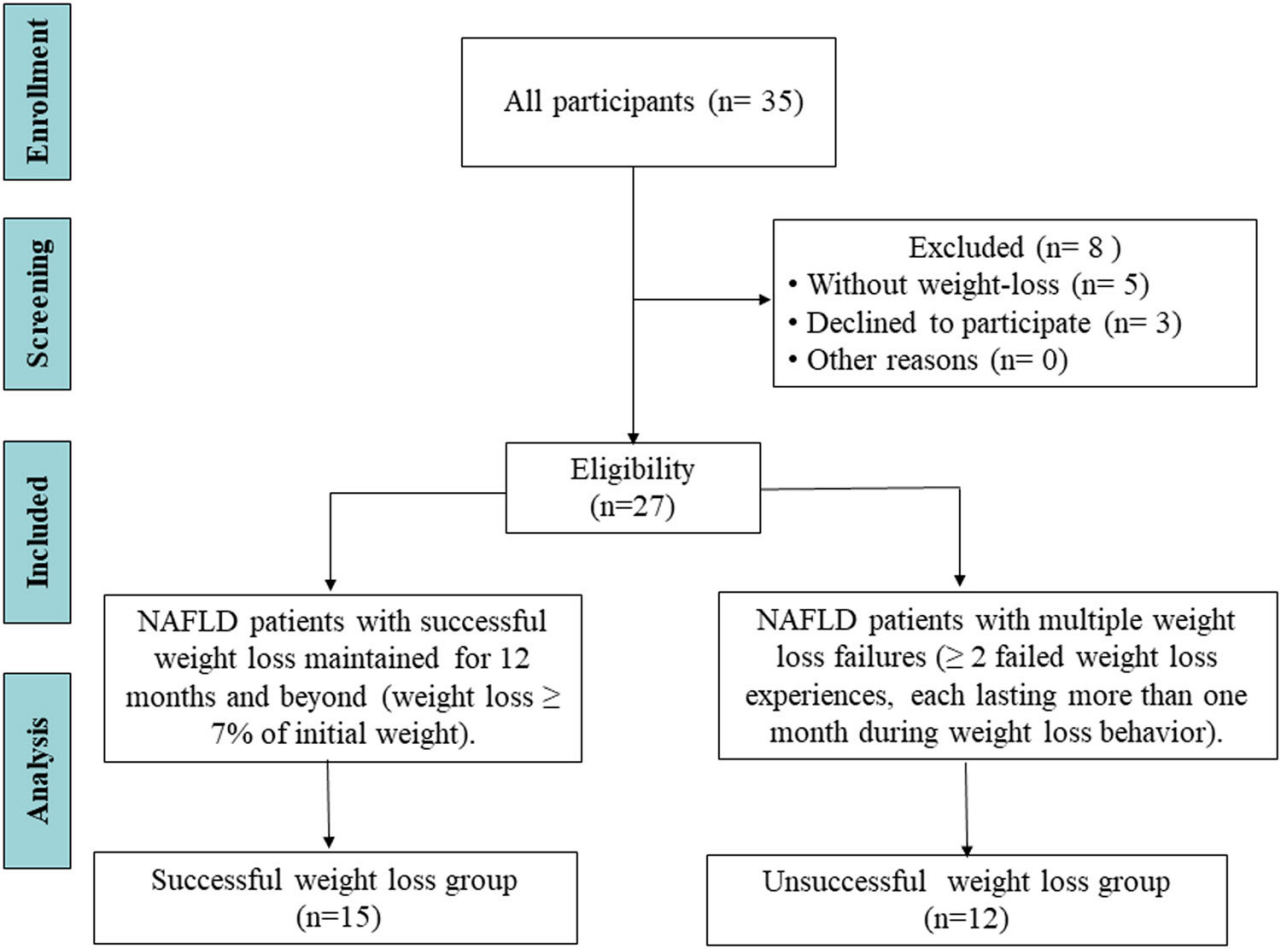
Flowchart of the screening process for the study participants

Two researchers (Y. G. and J. S.) recruited potential participants from the Bariatric Clinic, Hepatology Clinic and Physical Examination Center of the Affiliated Hospital of Hangzhou Normal University between March 2021 and October 2021. Researcher Y. G. conducted a simple interview with all the patients that included explaining the purpose, significance, content and informed consent of the study to the patients. The patients were asked whether they had planned weight loss activities or programmes. If they answered ‘yes’, they were then asked if their weight loss experience met the following inclusion criteria: (1) NAFLD patients with successful weight loss maintained for 12 months and beyond (weight loss ≥7% of initial weight); (2) NAFLD patients with multiple weight loss failures (≥2 failed weight loss experiences, each lasting more than 1 month during weight loss behaviour, including both those with repeated weight loss failures and those with successful but unmaintained weight loss). Patients with NAFLD without weight loss experience were excluded. Figure 1 shows the participant screening process. The sample size required was determined when saturation of themes was achieved.^19^

### 2.3 Data collection

Eligible participants were asked to participate in a 60‐min semistructured interview. The COM‐B model was used as a topic guide for interviews. All interviews were conducted by one of two chief researchers (Y. G., a practising physician and PhD candidate, and Y. H., a registered nurse with a PhD working in the university sector). Both were clinical fellows with qualitative research training. Written consent was obtained from participants who were able to complete an in‐person interview, and verbal consent was obtained for telephone interviews from participants who were unable to complete an in‐person interview due to logistics or distance. Patients were assured that they were free to withdraw at any time during the interview without any consequences. In addition, the researchers who conducted the interviews were not involved in providing care to the patients, so the patients' decision to participate did not affect their treatment. Interviews were conducted at a time and venue convenient to the patient. Participants were asked to describe their weight loss experiences. Upon completion of their interviews, participants were asked to complete a demographic survey, which included questions about their age, sex and socioeconomic status. The interviews were audio‐recorded, transcribed verbatim and returned to the participants for verification.

### 2.4 Data analysis

A combination of inductive content and deductive framework analysis using the COM‐B model was conducted.^20^ In the first stage of analysis, data were uploaded to NVivo v.11 software to facilitate data management, and then all transcripts were read repeatedly by the first author to become familiar with the whole data set. All transcripts were reviewed, and meaningful text was inductively coded by two authors. Five transcripts were read and coded by the other two authors to ensure reliability. Regular meetings among all authors occurred in which the independent coding was compared and discussed (the conference was moderated by the two main interviewers). This procedure continued until the interviewers agreed on a set of established codes. Once all codes were determined, the two main authors discussed and allocated each code to the appropriate component of the COM‐B model. Regular discussions took place during this process to ensure that all codes were allocated to the most appropriate COM‐B domain. Disagreements were formally resolved at each step by discussion and in consultation with two other investigators.

## 3 RESULTS

### 3.1 Study participant characteristics

Table 1 shows the characteristics of the participants. We obtained informed consent from and interviewed 27 participants: 15 participants who successfully achieved weight loss (SPs) and 12 participants who were unsuccessful in achieving weight loss (UPs).

**Table 1:** Participant characteristics (*n* = 27)

	SLWG (*n* = 15)	ULWG (*n* = 12)
Age (mean, SD)	39.27 (13.07)	39.50 (13.46)
Sex (*n*, %)		
Male	9 (60.0)	5 (41.67)
Female	6 (40.0)	7 (58.33)
Highest level of education (*n*, %)		
Graduate or postgraduate	9 (60.0)	3 (25)
Bachelor's degree (BA or BS)	3 (20.0)	6 (50)
High school graduate	3 (20.0)	3 (25)
Current work status (*n*, %)		
Working full‐time or part‐time	11 (73.33)	10 (83.33)
Student	2 (13.33)	1 (8.33)
Retired	2 (13.33)	1 (8.33)
Annual household income (*n*, %)		
High‐income	9 (60.0)	3 (25)
Middle‐income	4 (26.67)	6 (50)
Low‐income	2 (13.3)	3 (25)
Time of NAFLD diagnosis (years) (mean, SD)	3.4 (4.0)	6 (4.59)
Weight loss experiences (*n*) (mean, SD)	1.8 (0.56)	1.33 (1.16)
Baseline weight (kg) (mean, SD)	75.76 (12.69)	81.18 (17.12)
Current weight (kg) (mean, SD)	67.19 (10.02)	
Baseline BMI (kg/m^2^) (mean, SD)	26.88 (2.11)	28.62 (3.96)
Current BMI (kg/m^2^) (mean, SD)	23.89 (1.59)	
Body weight change (kg) (mean, SD)	8.57 (4.95)	
Weight loss time (months) (mean, SD)	3.6 (1.81)	
Weight maintenance time (years) (mean, SD)	3.73 (4.64)	

Abbreviations: BA, bachelor of Arts; BS, bachelor of Science; kg, kilogram; n, number; SLWG, successful weight loss group; ULWG, unsuccessful weight loss group.

### 3.2 Barriers and facilitators

Thirty‐five themes were mapped onto the COM‐B model as barriers to and facilitators of weight management, as described in full below. These barriers are summarized in Table 2.

**Table 2:** Identified barriers and facilitators in the various domains of the COM‐B

Item	Domain	Definition	Barrier	*N*	Facilitators	*N*
Capability	Psychological capability	Any mental process or skill that is required for the person to perform the behaviour	Lack of correct weight loss knowledge	4	Basic weight loss knowledge	9
			Lack of rational thinking	3	Rational thinking before taking weight loss action	5
					Autonomous and active learning ability	4
					Steady mindset	3
	Physical capability	Any set of physical actions that require an ability or proficiency learned through practice	Unbearable exercise‐related exhaustion	4	Basic weight loss skills	9
			Unsustainable starvation diets	3	Weight loss methods suitable for individuals	4
			Withdrawal reaction	3	Previous exercise habits	3
Motivation	Automatic motivation	Emotional responses, desires and habits resulting from associative learning and physiological states	Lack of awareness of weight	8	Focus on personal image	4
	Reflective motivation	Beliefs about what is good and bad, conscious intentions, decisions and plans	Lack of strong motivation for weight management	5	Strong motivation for weight management	9
			Lack of attention to NAFLD	6	Attention to NAFLD	6
			Treating food as a reward or compensation	6	Crisis awareness	4
			Unsatisfactory results after effort	4	Positive feedback from phased success	6
					Unsuccessful weight loss experiences	6
Opportunity	Social opportunity	Influences of friends, family, colleagues and other influential people that support the implementation or lack of implementation of a behaviour	Social entertainment	6	Healthy eating habits of family members	3
			Lack of social support	3	Family support	4
			Other people do not pay attention to NAFLD	3		
			Surrounding attitudes to weight loss	4		
			Unhealthy eating habits of family members	4		
			Attitudes and advice from medical personnel	4		
	Physical opportunity	Anything in the physical environment that discourages or encourages the performance of the behaviour	Lack of time and energy	12	Sufficient time and energy	5
			Easy takeaway fast food	4		
			Lack of available healthy meals at work	4		

Abbreviations: COM‐B, capability, opportunity, motivation, behaviour; NAFLD, nonalcoholic fatty liver disease.

#### 3.2.1 Key barriers and their associated COM‐B domains

##### Capability

###### Psychological capability

####### Lack of correct weight loss knowledge

Lack of correct weight loss knowledge, including diet and exercise, was a common hindering factor: ‘Due to the lack of correct knowledge, I have blindly tried some very popular weight loss methods, such as trying not to eat dinner and even extreme dieting, so it often ends in failure’ (UP7, female, aged 40 years).

####### Lack of rational thinking

Rational thinking before taking weight loss action includes a rational examination of the participants' motivation to lose weight, an expectation that difficulties may occur in the weight loss process and awareness of the time and energy needed. Many participants who failed to successfully lose weight showed a lack of rational thinking, and their weight loss actions tended to blindly follow trends rather than following objective thinking. They, therefore, tended to underestimate the difficulty of losing weight, and they easily gave up when they encountered unanticipated difficulties during weight loss: ‘There are tons of videos on Tik Tok or other social media that show how easy it is to lose weight … they claim that you can easily lose weight just by doing a few movements or eating certain foods, as they say … some of them are actually disguised advertisements for health products … It's easy to follow suit blindly if you don't have the ability to think rationally…’ (SP5, male, aged 30 years).

###### Physical capability

####### Unbearable exercise‐related exhaustion

Some participants said they did not like the weight loss method of exercise because exercise was too difficult: ‘Exercise is really too tiring. Every time I think of the feeling of being tired, I don't want to do it…. It is really too difficult for me’ (UP4, female, aged 34 years).

####### Unsustainable starvation diets

Many participants thought that a starvation diet may have an effect in the short term, but it was detrimental in the long term: ‘A starvation diet is an anti‐human instinct … and you need to spend a lot of energy to fight this feeling, which may make you unhappy…’ (SP4, female, aged 28 years).

####### Withdrawal reaction

Some participants said they faced a withdrawal reaction when they changed their dietary habits at the initial stage. ‘In the first two weeks of weight loss, I always want to eat something, even when I'm not hungry, I always feel like eating something…. I don't think this feeling is caused by hunger … I think it is more of an inertia…. Because of the habit of eating at any time and any where, it is uncomfortable to suddenly stop this behaviour’ (UP6, male, aged 50 years).

##### Motivation

###### Automatic motivation

####### Lack of awareness of weight

Most unsuccessful participants showed a lack of awareness of weight. Although many of them were already overweight, they still believed that they were ‘not so fat’ and their weight was ‘tolerable’. Even some middle‐aged and elderly people thought that losing too much weight was not conducive to health: ‘I am 173 cm and 83 kg weight. For me, too thin is not necessarily a good thing at my age. I think 80 kg is completely fine for me’ (UP2, male, aged 58 years).

###### Reflective motivation

####### Lack of strong motivation for weight management

Those who were unsuccessful in losing weight showed weaker motivation than those who lost weight successfully. Even if doctors told them they needed to lose weight, they had many reasons not to follow this advice: ‘Ultimately, I don't really think I need to lose weight’ (UP2, male, aged 58 years).

####### Lack of attention to NAFLD

The participants generally lacked an in‐depth understanding of NAFLD. In particular, a common belief among these UPs was that NAFLD has little effect on health, and some did not even consider NAFLD a disease: ‘I have diabetes and NAFLD. If you say that I need to lose weight because of NAFLD, I may not heed it, but if you tell me that I need to lose weight because of diabetes, I will certainly heed it because it (diabetes) is really a very serious disease…. I don't think NAFLD has any serious impact’ (UP2, male, aged 58 years).

####### Treating food as a reward or compensation

In both the successful and the unsuccessful weight loss groups, many participants mentioned that they treated food as a form of self‐reward or compensation: ‘After a tiring day of work, I want to eat some food to compensate myself. In fact, I am not so hungry but still want to eat a good meal’ (UP5, female, aged 30 years).

####### Unsatisfactory results after effort

‘If I don't see any changes in the first few weeks, I would get frustrated and even want to give up’ (UP7, female, aged 40 years). Even those who lost weight successfully reported that ‘it is a blow if the weight has not been reduced in the initial stage of weight loss’ (SP4, female, aged 28 years).

##### Opportunity

###### Social opportunity

####### Social entertainment

Most participants mentioned that social entertainment was not conducive to weight management. In further interviews, we received the following feedback. First, the time spent eating during social entertainment is significantly increased compared with normal eating. During that time, people unconsciously eat more food. Second, social entertainment dinners tend to be varied and high in calories, which can easily lead diners to eat more food than they really need. Third, in a social situation, it seems inappropriate not to eat when everyone else is eating. In addition, social meals are often accompanied by drinking behaviour, which might also lead to overeating. ‘We often eat barbecue or hot pot at parties, which are quite caloric … and it is easy to eat more while everyone eats’ (UP1, male, aged 32 years).

####### Lack of social support

Some unsuccessful participants indicated that they encountered external adverse evaluations when they took weight loss actions: ‘I just started the job, and I am still a novice. I ate working meals with superior physicians after surgery, and sometimes the director or head nurse said that I wasted food when I left too much food’ (UP11, male, aged 28 years). ‘When I was trying to lose weight, I really hated hearing people say, “Wow, you're eating so little…. Can you eat enough with such a small amount of food?” … These words always made me feel that I was weird and a misfit compared to other people’ (UP8, female, aged 28 years).

####### Other people do not pay attention to NAFLD

‘Ultimately, they still don't think NAFLD is dangerous … for example, when we drink at dinner, if you say you have high blood pressure and the doctor forbids you to drink, everyone will understand, but if you say you have fatty liver and cannot drink, everyone will laugh at you and make you drink more’ (UP2, male, aged 58 years).

####### Surrounding attitudes to weight loss

Many participants said that the attitude of the people around them affected them. In particular, when people around them did not think that it was necessary for them to lose weight, they tended to give up on weight loss: ‘I always complained to my husband that losing weight is so painful. He would tell me, “You are not so fat, stop losing weight, why make life so hard, enjoy food”…’ (UP3, female, aged 34 years).

####### Unhealthy eating habits of family members

‘My mother is in charge of cooking for the family every day. She has a strong taste and always cooks food that is too oily and salty. We've complained about it, but she wasn't happy, so no one ever mentioned it again’ (UP5, female, aged 30 years).

####### Attitudes and advice from medical personnel

Many participants identified attitudes and advice of medical staff as directly influencing their perceptions of NAFLD. Some participants said their doctors told them that NAFLD was not as severe as diabetes or hypertension, so they did not pay attention to NAFLD and did not urgently take weight loss actions. Others said their doctor's advice to lose weight was too general to act: ‘Every doctor will say that you have to lose weight and eat less and so on, but I think they just say it casually because their attitude does not seem to make me feel that the disease is serious’ (UP8, female, aged 28 years), or ‘Doctors told me that NAFLD would be better if I lost weight, but no one told me more details … such as how much diet to lose or how long to exercise’ (UP2, male, aged 58 years).

###### Physical opportunity

####### Lack of time and energy

Almost all participants indicated that lack of time and energy limited their weight loss and even led people who had already lost weight successfully to regain the weight: ‘I can maintain that (good) figure, but the premise of maintaining this figure is that you have enough time and energy to do this. In fact, I am now fat because I am too tired, and I have no time. If I had a month to spare, I could still lose weight successfully at the rate of one‐half kilogram a day’ (SP2, male, aged 31 years).

####### Easy takeaway fast food

Many participants said that convenient takeaway resources can affect weight loss: ‘Because no one limits my eating, I can eat what I want, I can eat when I want, and there are no restrictions…. Even late at night, I can still get delicious fast food if I want…’ (SP4, female, aged 28 years).

####### Lack of available healthy meals at work

For work reasons, many participants needed to eat in the workplace canteen or go to restaurants near their workplace for lunch, but most of these foods have ‘excessive carbohydrates, heavy oil and heavy salt, and are not healthy enough’ (UP6, male, aged 50 years).

#### 3.2.2 Key facilitators and their associated COM‐B domains

##### Capability

###### Psychological capability

####### Basic weight loss knowledge

Most successful participants had a correct basic knowledge of diet and exercise that they used in weight management, such as ‘eating more (low‐calorie) vegetables or fruits and less fat’. They emphasized gradually controlling and maintaining their weight by changing their dietary structure instead of ‘starvation’: ‘Mindless starvation is never a good idea … my experience of losing weight has taught me that changes in diet and necessary physical activity is the best way’ (SP2, male, aged 31 years).

####### Rational thinking before taking weight loss action

Most successful participants mentioned that ‘really wanting’ to lose weight was important when they talked about their motivation. After further questioning, they said that this ‘want’ involved not only ‘hope’ but also a rational examination of their motivation to lose weight, an expectation that difficulties may occur in the weight loss process, and an awareness of the time and energy needed: ‘Everyone will say that they want to lose weight … but many people jump into action without thinking clearly, so their insistence does not always last long … people would have a more rational attitude after mature thinking’ (SP4, female, aged 28 years).

####### Autonomous and active learning ability

Some successful participants showed high autonomous learning and active learning ability: ‘I browse the internet for experience and knowledge shared by professionals … sometimes I ask people who are successful in losing weight how they do it…. Active learning is necessary’ (SP2, male, aged 31 years).

####### Steady mindset

Most of the participants stated that when they accepted the mentality that ‘weight loss is not so fast’, they were less disappointed during weight loss. This mentality also made it easier for them to persist until they saw an effect rather than giving up because they did not see an effect at the initial stage of weight loss.

###### Physical capability

####### Basic weight loss skills

Basic weight loss skills are the simple basic skills needed to facilitate long‐term weight management, such as calorie counting, diet matching and exercise skills. Many participants who successfully lost weight had one or several basic weight loss skills; for example, they could make simple nutritional combinations and healthy meals for themselves. Significantly, those who maintained a good weight for a long time seemed to be good at applying these skills to their daily lives: ‘I find it useful to have a simple nutritious meal cooking skills or effective exercise skills … for example, know how to prevent sports injuries, how to reasonably arrange exercise time and type, how to make healthy weight loss meals quickly…’ (SP6, female, aged 26 years).

####### Weight loss methods suitable for individuals

Many successful participants emphasized the importance of weight loss methods that were suitable for themselves: ‘I have no way to decide what the cook does when I eat in the staff canteen, but I can rinse the oil away with warm water every time before I eat it’ (SP13, male, aged 64 years), or ‘I don't like running … so I replaced it with brisk walking … for example, I walk to take express deliveries … I make a conscious effort to increase my walking time every day’ (SP3, female, aged 34 years).

####### Previous exercise habits

A small number of successful participants felt that exercise was interesting because they had exercise habits in their childhood: ‘A lot of people I knew couldn't hold to a workout. I am very different from them because of my family education … my father often took me to participate in various sports exercises when I was young … such as playing basketball or swimming … so … I feel like this is relaxing for me’ (SP2, male, aged 31 years).

##### Motivation

###### Automatic motivation

####### Focus on personal image

Among the participants who maintained a good weight, many showed concern about their physical appearance. They could not accept their weight gain and wanted to maintain their ideal body shape rather than endure it: ‘A good appearance is not just a good social card, it also pleases me … I can wear all kinds of beautiful clothes and show my beauty … that makes me feel good’ (SP15, female, aged 35 years).

###### Reflective motivation

####### Strong motivation for weight management

Almost all successful participants had clear and strong motivation. A common motivation among the younger group was the pursuit of a beautiful appearance, while the common motivation among the older group was the pursuit of health. In addition, there were short‐term motivations or goals, such as work needs, marriage and fertility needs: ‘I didn't get pregnant for two years after my marriage because I was diagnosed with polycystic ovarian syndrome…. The doctor said my obesity and fatty liver would make it worse…. So, I had to lose weight’ (SP8, female, aged 31 years).

####### Attention to NAFLD

Compared with those who failed to lose weight, those who succeeded showed more attention to NAFLD: ‘My doctor told me that I had NAFLD and also told me that my situation would improve if I could lose some weight … I felt that it was necessary to pay attention to it, so I took 2 months to lose 3 kg’ (SP11, female, aged 40 years).

####### Crisis awareness

Some participants indicated that it was not difficult to lose weight because of their crisis awareness. They preferred to take early weight loss actions when they were not very fat (or had not been fat for very long): ‘I gained 5 kg a year after I got married, but when I realized that I was fat, I immediately realized that I couldn't put it off any longer … losing 5 kg was not particularly difficult for me’ (SP3, female, aged 34 years).

####### Positive feedback from phased success

Many participants mentioned the importance of positive feedback, especially achieving the desired results in the early stages of weight loss: ‘My blood pressure used to be very high and was not well controlled by taking antihypertensive drugs. Now (after losing weight successfully), my blood pressure is under control, and I feel relaxed. I really like this feeling’ (SP12, male, aged 59 years).

####### Unsuccessful weight loss experiences

Most of the participants had unsuccessful weight loss experiences, and many of the SPs mentioned unhealthy weight loss as a contributing factor: ‘I have tried many unhealthy methods and it hasn't worked … this has taught me that it is important to choose a healthy weight loss method that can be adhered to for a long time’ (SP7, male, aged 27 years).

##### Opportunity

###### Social opportunity

####### Healthy eating habits of family members

Some participants said that the eating habits of the person who cooked at home affected their diet: ‘My parents eat very light food. They love vegetables and eat less oil. It is much healthier for me to eat at home than outside, and it's easier for me to control my weight when I eat at home than at a restaurant’ (SP7, male, aged 27 years).

####### Family support

Many people who live with their families say that family attitudes are important. If their family members support their behaviour rather than discourage or even oppose it, they are more likely to carry out weight‐loss actions: ‘A lot of (Chinese) parents think it's ok for their children to be a little bit fat and even oppose their children losing weight. Fortunately, when I tell my parents I want to lose weight, they are very supportive. They agree with me that it is not good to be fat, so they support me in controlling my diet at home’ (SP4, female, aged 28 years).

###### Physical opportunity

####### Sufficient time and energy

Almost all participants, whether successful or not, mentioned the importance of sufficient time and energy. Most of their successful weight loss activities were completed in a relatively generous period of time: ‘My plan was carried out during summer vacation. I would like to say that weight loss absolutely needs time. You have to consider a lot of content, such as how to match your diet, how to perform exercise … there are so many details. You need enough time and energy to learn and to try to find suitable methods for yourself…’ (SP3, female, aged 34 years).

## 4 DISCUSSION

We applied the COM‐B model to improve our understanding of the barriers and enabling factors in the weight management of patients with NAFLD. This study shows that 19 barriers and 16 enabling factors contributed to the weight loss process among patients with NAFLD. We plan to use the identified barriers and enablers to promote weight management in healthcare among patients with NAFLD and to inform future interventions aimed at optimizing evidence‐based practice in NAFLD management since weight management is critical for the improvement of disease conditions.^8^, ^21^

### 4.1 Comparison with existing literature

Most of the findings of this study are consistent with the existing literature in confirming that patients with NAFLD/obesity experience a range of factors related to capabilities, opportunities and motivation that impact weight management during weight loss.^9^, ^12^, ^22^, ^23^, ^24^, ^25^, ^26^ Previous research has shown that patients often have an insufficient understanding of the disease, its progression, and its management.^22^ This is consistent with the ability factors (lack of knowledge, lack of attention to NAFLD) identified in this study as influencing weight loss by participants who failed to successfully lose weight. Strong personal motivation to lose weight (especially the pursuit of beauty and health) and positive feedback from phased success were also identified by participants in this study. This is consistent with the existing literature, which has found that the desire to achieve rapid weight loss to improve health and early and significant weight loss are both facilitators of engagement and adherence.^12^, ^23^ Participants in this study identified their psychological status as influencing their weight management. Many participants stated that stable mood states helped them control their weight, while others stated that negative psychological states (stress, anxiety, etc.) led to weight gain. This is consistent with existing research, which has identified psychological/emotional well‐being as an important factor in weight gain.^24^, ^25^ The participants in this study also identified social networks and support as an important influencing factor throughout weight reduction. This is consistent with prior research that identified social support as the most consistent facilitator of and barrier to lifestyle change.^12^ Participants in this study emphasized the importance of adequate time and energy for weight loss, especially in the initial stages. Many participants stated that their successful weight loss occurred over a sufficient period of time (e.g., winter or summer holidays), while others stated that working too much and being tired (lack of time and energy) made it difficult for them to adhere to lifestyle changes (e.g., adherence to exercise and a healthy diet). This is consistent with prior research that identified limited time and resources to support behaviour change.^26^ In addition, previous studies have shown that a lack of correct guidance and support from physicians is an important factor that affects lifestyle management in patients with NAFLD.^9^ This is consistent with factors related to medical staff that were identified in this study as influencing weight loss. Some participants stated they were told after their diagnosis of NAFLD that there was nothing concerning about NAFLD compared to other health conditions (e.g., diabetes or hypertension), and others said they lacked support to manage their condition effectively.

There are some differences between this study and the existing literature. Most strikingly, the participants in this study identified weight‐loss skills (e.g., diet matching, calorie counting, exercise skills) as important enabling factors for weight management in patients with NAFLD. Many participants stated that basic weight loss skills made it easier for them to lose weight successfully and maintain an ideal weight for a long period of time, while others stated that a lack of these basic skills made them unsure of how to do the right thing. Second, rational thinking before weight loss action was a highlighted factor identified in this study that has not been mentioned in previous literature. Many participants in this study who succeeded in losing weight stated that rational thinking was essential, including a rational examination of their motivation to lose weight, the expectation that difficulties may occur in the weight loss process, and an awareness of the time and energy required. This rational thinking helped them develop appropriate weight management goals and select appropriate weight loss methods to facilitate long‐term weight management. In addition, a focus on personal image and crisis awareness were identified in this study as important enabling factors by participants who successfully lost weight. They stated that they were unable to tolerate weight gain and tended to initiate weight loss activities early.

### 4.2 Clinical implications

NAFLD is not a sudden disease, and it often does not seem dangerous in the short term, which is why patients with NAFLD have a low willingness to receive advice from doctors on lifestyle management or weight control. This was reflected in our study. Among the patients in this study who successfully lost weight, only a few participants lost weight simply because of the emphasis on NAFLD; more participants adopted weight loss recommendations and took action because of multiple factors. This may suggest the following strategies for weight management recommendations to NAFLD patients. First, for patients with simple fatty liver, it is not sufficient to explain the long‐term harm of the disease. Many patients will not adopt advice to change their lifestyle, but they can be advised to lose weight from the perspective of personal image or general health. Second, for patients with metabolic diseases such as diabetes or hypertension, the harm of the disease should be emphasized because NAFLD often exacerbates their primary disease. This aggravation theory is more likely to arouse the attention of patients. Third, weight loss for NAFLD patients involves physical, psychological, nutritional, exercise and other aspects, which calls for multidisciplinary clinical approaches to help patients solve their problems. Finally, education on NAFLD should not be provided only to patients; because it is a lifestyle disease, family members living together should also understand its harm. The appropriate participation of family members can be considered in future education or interventions.

### 4.3 Strengths and limitations

The advantage of our research is that we interviewed successful and unsuccessful weight loss groups to increase the depth and credibility of the research. Furthermore, we used the purposive sampling method to include as many people as possible of different ages, genders, cultures and income levels so that the researchers could explore whether different themes appeared in these groups. However, our study also has some limitations. First, as a qualitative study, we could only preliminarily explore the problem and could not reveal causal relationships, which means that our results need to be confirmed by further quantitative studies with large samples. Second, some topics were not clearly classified; for example, social entertainment topics can be classified as both social opportunity and physical opportunity. To address this issue, we resorted to experts and reached an agreement through discussion among multiple authors. Third, we acknowledge that the volunteers who participated in the semistructured interviews were likely to be passionate about the topic. This is a common limitation of qualitative interviews that cannot be avoided.

## 5 CONCLUSION

This study identified a range of factors related to capability, opportunity and motivation, consistent with the existing literature. This study also identified factors such as basic weight loss skills and rational thinking before weight loss that influence weight management in patients with NAFLD that were not previously reported. This has clinical implications for clinical healthcare providers and health management services to improve education and support regarding lifestyle improvement and weight management in patients with NAFLD.

## AUTHOR CONTRIBUTIONS

Yunpeng Gu, Yanli Hu and Junping Shi conceived and designed the study. Yunpeng Gu and Yanli Hu developed study instruments, led the analysis and wrote the first draft of the manuscript. Yunpeng Gu, Yanli Hu, Wei Zhang, Yutong Chen contributed to data collection. Yunpeng Gu, Yanli Hu, Run Zhou and Tingting Kong coded the data, and Yunpeng Gu, Yanli Hu, Run Zhou and Tingting Kong contributed to the thematic analysis. Chunmei Wang contributed to the second round of manuscript revisions and a partial collection of materials. All authors had full access to all the study data and take responsibility for the data integrity and reliability of the analysis. All authors had final responsibility for the decision to submit for publication.

## CONFLICT OF INTEREST

The authors declare no conflict of interest.

## Supporting information

Supplementary Information

Supplementary Information

## Data Availability

The data that support the findings of this study are available from the corresponding author upon reasonable request.
