# Supplementary material for: Barriers and enabling factors in weight management of patients with nonalcoholic fatty liver disease: A qualitative study using the COM‐B model of behaviour
Source: Health Expect. 2022 Nov 16;26(1):355–65. doi: 10.1111/hex.13665 (PMC9854286; doi:10.1111/hex.13665)
Supplement: Supplementary file 2 — Supplementary Information [file HEX-26--s001.docx]

Additional file: Interview structure, including the original Chinese set of questions and the English translation.

| Original Chinese set of questions | English translation |
| --- | --- |
| 解释：  下划线标注：主要问题  灰色区域是给访谈者的提示  *斜体字表示对问题的补充和解释*   - 圆点：罗列的是备选问题（当受访者回答信息不足时可以使用主要问题下罗列的备选问题进行提问） | Explanation:  Underlined text: Main question  Grey fields are instructions for the interviewer  *Italics: connection or explanatory text*   - Listing: supplementary questions (to be used only if the spontaneous response has not contained any concrete information) |
| 介绍：致谢 预备  *我代表团队感谢您能参与本次研究访谈。本研究旨在改善NAFLD患者体重管理现状。*  *您向我们讲诉、分享您的真实减重体验对我们的研究会有很大的帮助。如果您愿意，访谈结束后我们会向您反馈结果。*  *为了不遗漏任何细节，请您允许我们录音。之后的录音转录均会以匿名形式进行，不会透露您的真实身份。* | Introduction: thanking, warm-up  *I would like to express the acknowledgement on behalf of our team and stress how grateful we are for your participation in this interview. The aim of the study is to improve the current status of weight management in patients with NAFLD.*  *The fact that you share your actual experience with us highly contributes to our study. If you wish, we will gladly inform you about the final results as soon as the follow-up has been completed.*  *I ask for your permission to make a voice recording, not to miss any of your comments. The transcription of the recording and the evaluation will be carried out in an anonymous form, and subsequently cannot be traced back to you.* |
| *本次访谈的目的是深入了解NAFLD患者体重管理的各个方面，您可以自由表达您的观点，即便您的观点不在问题调查范围内亦可。* | *The aim of this interview is to get a more sophisticated understanding about the aspects of weight management among NAFLD patients and to provide you the possibility to freely express your view which wasn`t covered by the questionnaire.* |
| 1. 问题 -引导受访者逐渐进入状态  您能不能先聊一聊您的体重增加的经过? | 1. Question - Guide the interviewee to enter the state gradually  Would you first tell me about your weight gain experience? |
| 2. 问题 -探究与本研究相关的经验  您能不能聊一聊您的减重经历? | 2. Question - Exploring the experiences concerning the study  Would you please tell me about your weight loss experience? |
| 如果需要的话可以询问以下问题来调动患者:   - 您一共有几次经历？（成功几次/不成功几次） - 您如何准备的？减重多少？维持多久？ - 成功和不成功的经历对比有何不同？ | Supplementary questions if needed for warming up:   - How many times have you been through this? How many times you successes? How many times you failed? - How did you prepared? How much weight did you loss? How long? - What is the difference between your successful and unsuccessful experiences? |
| 3. 问题 – 确定想法和行为之间的差距，以及造成这些差距的促进或阻碍因素  您觉得管理体重难不难或者容易吗?为什么？ | 3. Question - Identification of gaps between thought and behavior, and barriers or facilitators to reduce these gaps  How easy or difficult do you find it to manage your weight? Why? |
| 4. 问题 – 关于脂肪肝以及体重管理的知识和态度  您怎么看待脂肪肝患者管理体重? | 4. Question - Knowledge and attitudes regarding weight management and NAFLD  What is your opinion with weight management among NAFLD patients? |
| 如果回答信息不足，可以补充以下问题:   - 您怎么看到脂肪肝? - 您认为脂肪肝患者减重重要吗？ - 您希望从体重管理中获得什么？ | Additional questions if the answer was not exhaustive:   - What do you think about NAFLD? - Do you think weight loss is important for NAFLD patients? - What do you expect from weight management ? |
| 5. 问题 -探索影响减重实践的因素 (1): 能力  (具体包括:知识、科学原理、信念、记忆力、注意力、决策过程、程序性知识、行为调节、技能、自我效能)  您认为自己有能力管理好自己的体重吗? | 5. Question - Exploration of the factors influencing the weight loss implementation (1): Capability  (domains of these field: knowledge, scientific rationale, beliefs, memory, attention, decision process, procedural knowledge, behaviour regulation, skills, self-efficacy)  Do you think you would be able to manage your weight by yourself? |
| 如果回答信息不足，可以补充以下问题:   - 是什么鼓励您这么做? - 您怎样看待其中的困难? - 哪些外在环境对您减重有利？ - 您对减重的过程适应吗？为什么？ | Additional questions if the answer was not exhaustive:   - What would support or prevent you from doing so? - How do you estimate the difficulties of loss weight? Why? - What circumstances could support your weight loss implementation? - Are you satisfied with the process of loss weght? Why? |
| 6. 问题 – 探索影响减重实践的因素 (2): 机会  (具体包括: 时间、资源、地理位置、诱因、物质等物理环境；文字、观念、人际影响、社会文化习俗等社会环境)  您如何评价您减肥过程中的各种机会?为什么？什么是您最需要的资源或者最主要的影响因素？ | 6 Question - Exploration of the factors influencing the weight loss implementation (2): Opportunity  (domains of this field: time, resources, geographical location, inducement, material and other physical environment; characters, ideas, interpersonal influence, social and cultural customs and other social environment)  How do you rate the chance in your weight loss implementation? Why? What are the main influencing factors and required resources? |
| 如果回答信息不足，可以补充以下问题:   - 您的朋友、家庭、同事或者其他人对您有什么影响吗？ - 您的文化背景（信念、价值观等）会影响您的减重行为吗？ - 这些社交或者专业影响多大程度上阻碍或者促进了您的减重行为呢？ - 谁的意见对您减重行为的影响最大？ - 这些对您影响的人如何看待减重？ - 您感觉以上各个方面影响到您的减重行为了吗？为什么？ | Additional question if the answer was not exhaustive:   - What about the influence of your friends, family, colleagues or someone else? - How do you assess the impact of your cultural background (general beliefs, values, assumptions) regarding the implementation of weight loss? - To what extent do these social/professional influences facilitate or hinder your weight loss implementation? - Whose opinion do you think has had the most influence on your weight loss behavior? - What do these people who influence you think about weight loss? - Do you feel that any of the above aspects are relevant to you and your weight loss behavior? Why? |
| 7. 问题 – 探索影响减重实践的因素(3): 动机  (具体包括:联想学习产生的情感反应、欲望、习惯以及生理状态;对什么是好的和坏的信念，有意识的意图，决定和计划)  减重与您的工作/自身状况相符合还是相冲突? | Question 7 - Exploring the factors influencing the weight loss implementation (3): Motivation  (domains of these field: emotional responses, desires and habits resulting from associative learning and physiological states; beliefs about what is good and bad, conscious intentions, decisions and plans)  Is losing weight compatible or in conflict with your professional standards/identity? |
| 如果回答信息不足，可以补充以下问题:   - 您对此有什么感受？ - 您感觉在减肥过程中有什么困难吗？ - 您根据减肥对您有好处还是坏处？ - 您发自内心真的想减肥吗？ | Additional question if the answer was not exhaustive:   - What feelings do you have in this regard? - Do you anticipate any problems with weight loss implementation? - What are the benefits and disadvantages of weight loss implementation for you? - Do you really want to lose weight from your heart? |
| *非常感谢您的配合，我已经完成所有的问题了，您还有什么想要了解或者需要补充的吗？*  *再次感谢您给我这次访谈机会，感谢您给我分享自己真实的体验和感受！* | *Thank you very much for your cooperation. I have finished all the questions. Is there anything else you want to know or need to add?*  *Thank you again for giving me this opportunity to interview, thank you for sharing your real experience and feelings with me.* |
